# Patient-Reported Outcome Measures for care quality assessment of a breast cancer Care Pathway

**DOI:** 10.1093/eurpub/ckac131.293

**Published:** 2022-10-25

**Authors:** C Angioletti, E de Mattia, A Perilli, AG de Belvis

**Affiliations:** 1 Department of Life Sciences and Public Health, Università Cattolica del Sacro Cuore, Rome, Italy; 2 Critical Pathway and Outcome Evaluation Unit, Fondazione Policlinico University A. Gemelli IRCCS, Rome, Italy

## Abstract

**Background:**

Breast Cancer (BC) is the most common type of cancer among women in Europe, accounting for around 28% of newly diagnosed female cancers. A clinical Pathway (CP) is an effective tool able to deliver high quality care especially if linked to a Monitoring System. Still, it is essential to integrate them with the patient’s perspective according to Value-Based Care. This paper aims to define and test a tool for care quality assessment in patients with breast cancer, in a value-based perspective.

**Methods:**

A scoping search of the main databases (PubMed, Scopus, Web of Science) and official websites of institutions and organizations (AIOM, CIPOMO, EORTC, ICHOM, Istat) was carried out. A Delphi survey was conducted to assess the tool, according to four criteria (general relevance, evidence-based, measurability, actionability). We only included indicators that achieved strong agreement. Time-points for data collection were defined and validated in relation to the different steps of the CP.

**Results:**

The final tool consists of 21 questions coming from the following sources: BREAST-Q and EORT QLQ-BR23 questionnaire. BREAST-Q’s questions, have to be administered at T0 (first medical contact) and T12 (follow-up), while the EORT QLQ-BR23’s questions have to be administered at T0 (first medical contact) T Surgery, T6 (follow-up) and T12 (follow-up). The survey will be administered to a sample of 152 BC patients (MOE 5%; CL 95%) undergoing surgery in the period June-September 2021.

**Conclusions:**

The present tool will give a quick view of Value provided by the CP to these patients. In light of the high volume of patients treated at our center (1008 breast cancer surgery hospitalisations in 2020), this study acquires further public health relevance. Additionally, this approach can be extended for further evaluation of other CPs.

**Key messages:**

• We assess care quality for Breast Cancer patients in a large hospital in Italy through Patient-Reported Outcome Measures, based on an evidence-based, Value-based, Delphi-validated tool.

• Assessing Value brought to patients with Breast Cancer gives a unique perspective on care quality assessment and paves the way for further quality improvements and extension to other health issues.

